# Liuwei dihuang decoction attenuates intervertebral disc degeneration by inhibiting TRPA1-Mediated ferroptosis in endplate chondrocytes

**DOI:** 10.3389/fcell.2026.1759798

**Published:** 2026-05-29

**Authors:** Rui Xu, Yiwen Yang, Pengchao Xu, Ken Qin, Ouye Li, Tianlong Wu, Yibo Pan, Cenzhuo Sheng, Zhefei Xie, Mo Wu, Jinglei Wang, Weixiang Wang, Tianyou Ma, Zhaokai Jin, Dongqing Jiang, Hanting Xia, Yong Li

**Affiliations:** 1 Department of spine surgery, Affiliated hospital of Jiangxi University of Chinese Medicine, Nanchang, Jiangxi, China; 2 Zhejiang Provincial Chinese Medicine Hospital (First affiliated hospital of Zhejiang Chinese Medical University), Zhejiang Chinese Medicine University, Hangzhou, Zhejiang, China; 3 Department of Operations Management, Affiliated Hospital of Jiangxi University of Chinese Medicine, Nanchang, Jiangxi, China; 4 State Key Laboratory of Food Science and Resources, China-Canada Joint Lab of Food Science and Technology (Nanchang), Nanchang University, Nanchang, Jiangxi, China; 5 Jiangxi Institute of Quality and Standardization, Nanchang, Jiangxi, China; 6 Department of Pharmaceutical Engineering, School of Biomedical Sciences, Daegu Catholic University, Gyeongsan-si, South Korea; 7 Jiangxi University of Chinese Medicine, Nanchang, Jiangxi, China

**Keywords:** endplate chondrocytes, ferroptosis, intervertebral disc degeneration, traditional Chinese medicine formula, TRPA1-mediated ferroptosis

## Abstract

**Introduction:**

Liuwei Dihuang Decoction (LWDHD) is a well-known traditional Chinese medicine formula, which occupies an important position in clinical TCM practice. This study aimed to elucidate the therapeutic mechanism of LWDHD against Intervertebral Disc Degeneration(IVDD), with a specific focus on its role in preventing endplate chondrocyte degeneration and its interaction with the TRPA1-ferroptosis axis.

**Methods:**

The components of LWDHD were first characterized using high-performance liquid chromatography (HPLC). A mouse model of lumbar spinal instability (LSI) was then established to evaluate the therapeutic effects of LWDHD on IVDD-related pain, behavioral function, and intervertebral disc structure. Furthermore, an IL-1β-induced chondrocyte inflammation model was utilized to assess the impact of LWDHD on chondrocyte proliferation, lipid accumulation, oxidative stress, and ferroptosis. Based on predictions from network pharmacology and molecular docking regarding the key mechanisms of LWDHD against IVDD, core signaling pathways were experimentally validated using specific pharmacological agonists.

**Results:**

HPLC-MS analysis identified five representative compounds in LWDHD under both positive and negative ion modes. In a mouse model of LSI, LWDHD administration significantly alleviated discogenic pain and restored motor function. Micro-CT results demonstrated that LWDHD treatment preserved intervertebral disc height, protected the endplate against fibrotic structural degeneration, and reduced ROS accumulation, thereby attenuating ferroptosis-related tissue injury. Correspondingly, in IL-1β-induced chondrocytes, LWDHD suppressed intracellular lipid accumulation and oxidative stress, while also restoring chondrocyte proliferative capacity and upregulating the expression of the ferroptosis-related protein GPX4. Through network pharmacology and molecular docking, TRPA1 was identified as a potential therapeutic target of LWDHD. This finding was further validated by fluorescence co-localization and pharmacological activation, which confirmed the regulatory role of TRPA1 on GPX4. Importantly, the protective effects of LWDHD were abolished upon TRPA1 activation, underscoring its essential role in the observed therapeutic outcomes.

**Discussion:**

Our study demonstrates that LWDHD mitigates intervertebral disc degeneration by inhibiting TRPA1, thereby restoring redox homeostasis, preserving ECM integrity, and alleviating pain. This work validates the TRPA1-ferroptosis axis as a key pathogenic driver of IVDD and establishes a mechanistic basis for the evidence-based use and modernization of LWDHD in the treatment of degenerative musculoskeletal diseases.

## Graphical Abstract

**Figure:**
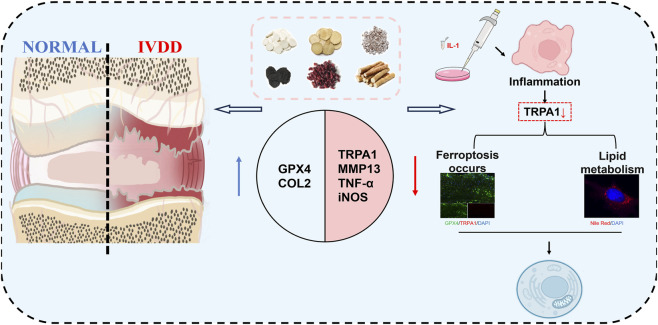

## 1 Introduction

Intervertebral disc degeneration (IVDD) is a principal cause of chronic low back pain and disability (Jing et al., 2024; Mohd Isa, Teoh, Mohd Nor and Mokhtar, 2022; Oichi, Taniguchi, Oshima, Tanaka and Saito, 2020). As a progressive and multifactorial disorder, IVDD features extracellular matrix (ECM) degradation, chronic inflammation, and gradual loss of disc-resident cells, together compromising biomechanical function and tissue integrity (Wang et al., 2025; Wang et al., 2023). Although degeneration of the nucleus pulposus has long been emphasized, emerging evidence highlights endplate chondrocytes (EPCs) as indispensable regulators of disc homeostasis (Bing et al., 2024; Shi et al., 2024). The cartilaginous endplate is the primary gateway for nutrient diffusion and metabolic exchange; EPC dysfunction disrupts this interface, amplifies oxidative stress, and accelerates degenerative cascades. Consequently, preventing EPC loss and dysfunction has become a rational therapeutic objective, yet pharmacological strategies that specifically protect EPCs remain limited (Wang et al., 2020).

Liuwei Dihuang Decoction (LWDHD), a classical traditional Chinese medicine formula composed of *Radix rehmanniae rraeparata, CornusofficinalisSieb. et Zucc, Dioscoreae Rhizoma, Moutan Cortex, Alismatis Rhizoma* and *Poria,* has been used for centuries to “tonify the kidney and nourish Yin,” a notion historically linked to spinal and skeletal health (Lu, Huang, Lin, Wang and Li, 2022; Song et al., 2022). Contemporary pharmacology attributes to LWDHD anti-inflammatory, antioxidant, and anti-aging activities, suggesting potential value in degenerative musculoskeletal conditions. However, its cellular and molecular mechanisms in IVDD—particularly with respect to EPC preservation—remain poorly defined.

Among regulated cell-death modalities implicated in disc pathology, ferroptosis has recently emerged as a crucial driver of EPC depletion (Yang et al., 2021; Zhou L. P. et al., 2022). Ferroptosis is an iron-dependent process characterized by glutathione depletion, inactivation of glutathione peroxidase 4 (GPX4), and accumulation of lipid reactive oxygen species (ROS), culminating in membrane damage and ECM breakdown (Rochette et al., 2022; Wang et al., 2022). In parallel, the transient receptor potential ankyrin 1 (TRPA1) channel—a Ca^2+^-permeable sensor activated by electrophiles and oxidative stress—has been implicated in amplifying ROS signaling and ferroptotic susceptibility in multiple tissues. Whether TRPA1 governs ferroptosis in EPCs and thereby contributes to IVDD, however, remains unclear (Hu et al., 2023; Weng et al., 2024; Ye L. et al., 2025).

We therefore hypothesized that LWDHD mitigates IVDD by suppressing TRPA1-mediated ferroptosis in EPCs. To test this, we combined high-performance liquid chromatography–mass spectrometry (HPLC–MS) to define LWDHD’s chemical profile with network pharmacology and molecular docking to nominate TRPA1 as a candidate target. We then evaluated LWDHD in IL-1β(10 ng/mL)–stimulated primary EPCs *in vitro* and in a puncture-induced IVDD mouse model *in vivo* (Xue et al., 2021). Finally, we performed a pharmacological rescue experiment using cinnamaldehyde (hereafter referred to as Agonist-1 in the text and figures andthe concentration used was 2 μL/g), a selective agonist of TRPA1, to determine whether activation of TRPA1 could abolish the protective effects induced by LWDHD (Dou et al., 2025; Feng et al., 2025; Mori and Urata, 2024). Our findings indicate that LWDHD alleviates pain-related behaviors, preserves endplate structure, restores ECM homeostasis, and limits ferroptotic stress in EPCs, and that these effects require modulation of TRPA1 signaling.

## 2 Materials and methods

### 2.1 Preparation of LWDHD and LWDHD -containing serum

LWDHD is a traditional Chinese medicine formula composed of the following herbs: *Rehmannia glutinosa* (Gaertn.) DC.*, Cornus officinalis* Siebold & Zucc., *Dioscorea opposita* Thunb.*, Alisma plantago-aquatica* L., *Paeonia × suffruticosa* Andrews, *Wolfiporia cocos* (F.A. Wolf) Ryvarden & Gilb. (All botanical plant names above have been verified through http://www.worldfloraonline.org. And the date of accessing the website is 16 October 2025.) The composition of LWDHD, including herbal species, medicinal parts, origins, and dosages, is summarized in Table 1. All herbs were obtained from the First Affiliated Hospital of Zhejiang Chinese Medical University (Hangzhou, China). The herbal mixture was soaked in water (four times its volume) and subsequently decocted. The resulting decoction was filtered and concentrated to a final volume of 100 mL using a rotary evaporator at 55 °C. The concentrate was then lyophilized in a freeze dryer to obtain a lyophilized powder, which was stored at −40 °C. The lyophilized powder was reconstituted with ddH_2_O. Based on body surface area conversion, the administered concentrations of the crude drug for the low, medium, and high-dose groups were set at 0.1725 g/mL, 0.345 g/mL, and 0.69 g/mL, respectively. The solution was administered via gavage at a dosage volume of 0.3 mL per 20 g of body weight.

**TABLE 1:** Composition and origin of LWDHD.

Pharmaceutical name	*Botanical plant names* and authorities	Traditional medicinal parts	Origin	Weight (g)
Shu dihuang	*Rehmannia glutinosa* (gaertn.) DC.	Root tuber	Henan	15
Shan zhuyurou	*Cornus officinalis* siebold & zucc	Pulp	Zhejiang	12
Shanyao	*Dioscorea polystachya* turcz	Root tuber	Zhejiang	12
Mu danpi	*Paeonia × suffruticosa* andrews	Root bark	Anhui	10
Zexie	*Alisma plantago-aquatica* L	Root tuber	Sichuang	10
Fuling	*Wolfiporia cocos* (F.A. Wolf) ryvarden & gilb	Sclerotia	Zhejiang	10

### 2.2 HPLC analysis

The LWDHD extract was analyzed using an Agilent 1260 HPLC system (Agilent, Waldbronn, Germany). Separation was performed on a Waters Cortecs UPLC T3 column (2.1 mm × 100 mm, 1.6 μm) maintained at 35 °C. The mobile phase consisted of solvent A (pure water) and solvent B (acetonitrile). The gradient elution program was set as follows: 78% A and 22% B from 0 to 14 min; a linear gradient from 22% to 30% B between 14 and 15 min; maintained at 30% B from 15 to 25 min; followed by a linear gradient from 30% to 60% B from 25 to 28 min; and finally, 60% B was held for an additional 10 min. The flow rate was 1.0 mL/min, and the injection volume was 20 μL.

### 2.3 Molecular docking

The three-dimensional (3D) crystal structure of the target protein was retrieved from the Protein Data Bank (PDB) database. The 3D structures of the candidate small-molecule ligands were downloaded from the PubChem database in SDF or MOL2 format and converted into PDBQT format after structural optimization. Molecular docking was performed using AutoDock. The docking parameters were set based on the active binding pocket of the target protein, and docking calculations were then carried out. The docking conformations were ranked according to their binding energies, and the conformation with the lowest binding energy was selected as the optimal binding pose. The ligand–protein interaction complex was finally visualized using PyMOL.

### 2.4 Cell culture and cell viability assay

Chondrocytes were cultured in DMEM/F12 medium (CellMax, Beijing, China) supplemented with 10% fetal bovine serum and 1% penicillin-streptomycin-amphotericin B solution. Cell viability was assessed using a Cell Counting Kit-8 (CCK-8) (Bioss, Beijing, China). Briefly, chondrocytes were seeded in 96-well plates at a density of 4 × 10^4^ cells per well and allowed to adhere. Subsequently, the cells were treated with complete medium containing various concentrations of LWDHD lyophilized powder for 24, 48, and 72 h. Following treatment, a 10% CCK-8 solution was added to each well, and the plates were incubated at 37 °C with 5% CO_2_ for 2 h. The absorbance was measured at 450 nm and 600 nm using a multimode microplate reader (PerkinElmer, United States of America).

### 2.5 5-Ethynyl-2′-deoxyuridine (EdU) proliferation assay

Chondrocytes were seeded in 24-well plates at an appropriate density. After overnight culture for cell attachment and stabilization, the cells were subjected to the designated drug treatments. Following treatment, the EdU working solution was added to each well and the plates were incubated for 2 h at 37 °C. The medium was then aspirated, and the cells were fixed with 4% paraformaldehyde for 15 min at room temperature. After fixation, the cells were washed three times (5 min per wash) with the provided washing buffer. Permeabilization was performed using 0.3% Triton X-100 for 15 min at room temperature, followed by another wash cycle. A freshly prepared Click reaction solution was added to each well to completely cover the cells, and the plates were incubated in the dark for 30 min. After the reaction, the solution was removed, and the cells were washed again. Finally, the cell nuclei were counterstained with Hoechst 33,342. Images were acquired using a high-content screening system and analyzed with ImageJ software.

### 2.6 Nile Red staining for lipid detection

Cells were plated at an appropriate density in confocal dishes. After the treatments, the culture medium was removed, and the cells were gently rinsed twice with 1× PBS (30 s per rinse). The cells were then fixed with a sufficient amount of fixative solution for 15 min. After fixation, the solution was aspirated, and the cells were washed as described above. An appropriate amount of Nile Red staining working solution was added to cover the cells, followed by incubation in the dark at room temperature for 10 min. The cells were subsequently washed again. After the final wash, a small amount of PBS was retained to prevent the cells from drying out. Fluorescence images were captured using a laser scanning confocal microscope and analyzed with ImageJ.

### 2.7 2′,7′-dichlorofluorescin diacetate (DCFH-DA) intracellular ROS detection assay

Chondrocytes were seeded in 24-well plates. After an overnight attachment period and subsequent drug interventions, the original culture medium was removed. The cells were then incubated with an adequate amount of DCFH-DA detection solution (diluted in serum-free medium according to the manufacturer’s instructions) at 37 °C for 20 min. Following incubation, the staining solution was aspirated, and the cells were gently washed with PBS to remove excess probe. Fresh complete medium was added to the wells. Intracellular ROS levels were immediately quantified using a high content screening. The acquired images were analyzed using ImageJ software.

### 2.8 Western blotting

Chondrocytes were lysed using RIPA lysis buffer (CWBIO, Jiangsu, China) supplemented with a protease inhibitor cocktail (CWBIO, Jiangsu, China) according to the manufacturers’ instructions. The protein concentration was quantified using a BCA Protein Assay Kit (Beyotime, Shanghai, China). Proteins were separated by electrophoresis on polyacrylamide gels prepared with a PAGE Gel Fast Preparation Kit (Epizyme, Shanghai, China) and then transferred onto PVDF membranes (Millipore, United States of America). The membranes were blocked with 5% skim milk for 1 h at room temperature, followed by incubation overnight at 4 °C with primary antibodies against GAPDH, COL2, MMP13, and GPX4. After washing, the membranes were incubated with appropriate horseradish peroxidase (HRP)-conjugated secondary antibodies for 1 h at room temperature. Protein bands were visualized using a protein imaging system (ProteinSimple, United States of America). The antibodies used in this study are shown in Supplementary Table S1.

### 2.9 Quantitative real-time PCR (RT-qPCR)

Total RNA was isolated from chondrocytes using a commercial RNA extraction kit (Accurate Biology, China). The purity and concentration of the extracted RNA were determined using a NanoDrop spectrophotometer (Thermo Fisher Scientific, United States of America). Subsequently, complementary DNA (cDNA) was synthesized from total RNA with the PrimeScript RT reagent kit (TaKaRa Biotechnology, Japan). RT-qPCR was performed on a StepOnePlus Real-Time PCR system (Applied Biosystems, United States of America) using SYBR Premix Ex Taq (TaKaRa Biotechnology). The mRNA expression levels of COL2A1, MMP13, GPX4, TNF-α, and iNOS were analyzed, with GAPDH serving as the reference gene. Relative gene expression was calculated using the 2^−ΔΔCT^ method. All primer sequences used in this study are listed in Supplementary Table S2.

### 2.10 Experimental mice

In this study, 10-week-old C57BL/6 male mice (Shanghai Bikai Keyi Biotechnology, China) and housed under standard conditions with free access to food and water. The model was established after a period of adaptive feeding. The experimental protocol was approved by the Animal Experiment Ethics Committee of Zhejiang University of Traditional Chinese Medicine (Approval No: IACUC-20230925–08).

### 2.11 Abnormal stress-induced lumbar spine instability (LSI) mouse model

Randomly and evenly assign mice with similar body mass to each group.Mice were anesthetized via intraperitoneal injection of Zoletil at a dosage of 0.06 mL per 10 g of body weight and positioned in a prone position. Following dorsal hair removal and skin disinfection with alcohol, a longitudinal incision was made along the midline of the spine. The fascia was bluntly dissected to expose the bilateral erector spinae muscles, which were then separated axially. In some cases, a portion of the muscles was resected to fully expose the lumbar spinous processes (L3-L5). The exposed spinous processes were completely excised, and the supraspinal and interspinous ligaments were removed. The surgical site was disinfected with alcohol, and the abdominal cavity was inspected to confirm the absence of visceral bleeding and peritoneal integrity. The wound was subsequently closed by suturing the muscle, fascia, and skin in layers. Topical Gentamycin hydrochloride was applied to the incision site to prevent infection. In the Sham group, the same surgical procedure was performed, including the exposure of the spine and separation of the erector spinae muscles, but without the excision of the spinous processes or ligaments.

### 2.12 Thermal pain sensitivity test

Thermal pain sensitivity was assessed using the YLS-6B smart hot plate instrument (Shanghai Precision Instrument, China). The plate temperature was maintained at 50 °C. Each mouse was placed on the heated surface, and the latency to hind paw lifting or licking was recorded as the response indicator.

### 2.13 Gait analysis

Mouse gait was recorded and analyzed using a DigiGait imaging system (Mouse Specifics, United States of America). Mice were allowed to run adaptively on a treadmill set at a constant speed of 18 cm/s. Gait parameters were subsequently collected and quantified using ventral plane imaging technology.

### 2.14 Histology and Micro-CT scanning

Following euthanasia, the L3-L5 spinal segments were dissected for histopathological examination. The isolated spine samples were initially scanned and analyzed using a Skyscan 1176 micro-CT system (Bruker micro-CT, Belgium). Subsequently, the samples were fixed in 4% paraformaldehyde (Solarbio, China) for 48 h. After fixation, decalcification was performed using a 14% EDTA solution (Sangon Biotech, China). The tissues were then washed under running water, dehydrated through a graded ethanol series, cleared, and embedded in paraffin. Serial sections were cut at a thickness of 4 μm for subsequent staining.

### 2.15 Immunohistochemistry (IHC) and immunofluorescence (IF)

For IHC, the deparaffinized and rehydrated tissue sections underwent antigen retrieval. Endogenous peroxidase activity was blocked by incubation with a peroxidase blocker for 15 min. The sections were then incubated with primary antibodies at 4 °C overnight in a humidified chamber. The following day, the sections were incubated with a biotin-labeled goat anti-rabbit IgG polymer for 15 min, followed by horseradish enzyme-labeled streptavidin working solution for another 15 min. Color development was achieved using a DAB substrate for 30 s. Finally, the nuclei were counterstained with hematoxylin, and the sections were differentiated in 1% acid alcohol, dehydrated, cleared, and mounted with a neutral resin.

For IF staining, after antigen retrieval and blocking, the sections were incubated with the respective primary antibodies overnight at 4 °C, followed by incubation with species-matched fluorescent secondary antibodies. The sections were then mounted with an anti-fade mounting medium containing DAPI. The antibodies used in this study are shown in Supplementary Table S1.

### 2.16 Alcian blue/hemotoxylin & orange G (ABH/OG) staining

Selected tissue sections were subjected to ABH/OG staining (Sigma, United States of America). The degree of cartilage degeneration was assessed using the histological endplate grading score.

### 2.17 Dihydroethidium (DHE) staining

After deparaffinization and rehydration, tissue sections were covered with 200 µL of washing solution and incubated at room temperature for 3–5 min. The solution was carefully removed, and an appropriate amount of staining probe working solution (BB-470516) was applied. The sections were then incubated at 37 °C in the dark for 60 min Following incubation, the staining solution was aspirated, and the sections were washed three times with PBS (3 min per wash). Nuclei were counterstained using an anti-fade mounting medium containing DAPI. Fluorescence images were acquired using a slide scanner (OLYMPUS, Japan) and analyzed with ImageJ software.

### 2.18 Statistical analysis

All statistical analyses were performed using SPSS 25.0 software. Measurement data conforming to a normal distribution are expressed as the mean ± standard deviation (x̅ ± s). Data that did not follow a normal distribution were analyzed using nonparametric tests. Correlation analyses were conducted using Pearson’s correlation coefficient and curve fitting. A two-tailed test level of α = 0.05 was adopted, and a P-value <0.05 was considered statistically significant.

## 3 Results

### 3.1 HPLC–MS analysis reveals abundant chemical constituents of LWDHD

**FIGURE 1:**
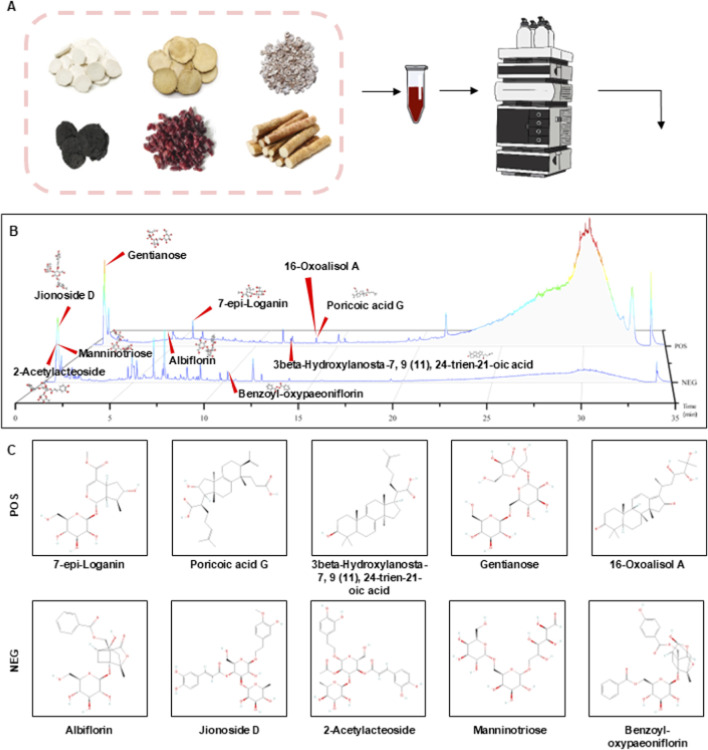
Experimental workflow and HPLC–MS profiling of lyophilized LWDHD powder. **(A)** Schematic overview of the analytical workflow. Lyophilized powder of Liuwei Dihuang Decoction (LWDHD) was reconstituted and subjected to high-performance liquid chromatography–mass spectrometry (HPLC–MS) for non-targeted profiling. **(B)** Representative total ion chromatograms (TICs) of LWDHD acquired in positive and negative ionization modes. **(C)** Identification of the top five representative constituents detected in each mode, annotated based on retention time, precursor ions, and diagnostic MS/MS fragmentation characteristics.

To characterize the chemical profile of LWDHD, the aqueous decoction was prepared according to standard procedures, lyophilized into powder, and subsequently reconstituted for high-performance liquid chromatography–mass spectrometry (HPLC–MS) analysis. The experimental workflow is illustrated in Figure 1A. Both positive and negative ionization modes were employed to comprehensively profile the chemical constituents.

The resulting Total Ion Chromatograms (TICs) displayed complex and well-resolved chromatographic patterns in both ionization modes, reflecting the diverse chemical composition of LWDHD (Figure 1B). Based on relative ion intensities, the five most abundant compounds in each mode were selected as representative constituents (Figure 1C).

In the positive ion mode, the predominant compounds included 7-epi-loganin, poricoic acid G, 3β-hydroxylanosta-7,9(11),24-trien-21-oic acid, gentianose, and 16-oxoalisol A. In the negative ion mode, the top five compounds comprised albiflorin, jionoside D, 2-acetylacteoside, manninotriose, and benzoyl-oxypaeoniflorin. The detailed HPLC–MS parameters of these ten representative metabolites—including compound name, retention time (RT), molecular formula, m/z values, ion response, and adduct type—are provided in Table 2. These molecules represent diverse structural classes such as iridoid glycosides, triterpenoid acids, phenylethanoid glycosides, and oligosaccharides, consistent with the major herbal components of the decoction.

**TABLE 2:** The detailed HPLC-MS parameters of representative compounds.

Peak No.	Observed RT(min)	Component name	Formula	Observed m/z	Response	Adduct
1	6.15	7-Epi-loganin	C17H26O10	413.1433	6,001	+Na, +H, +K
2	13.7	Poricoic acid G	C30H46O5	487.3426	6,515	+H
3	12.16	3beta-hydroxylanosta-7,9 (11),24-trien-21-oic acid	C30H46O3	455.353	6,030	+H
4	0.78	Gentianose	C18H32O16	505.1783	5,072	+H
5	13.7	16-Oxoalisol A	C30H48O6	505.3525	35,617	+H, +Na
6	6.75	Albiflorin	C23H28O11	525.1595	259,628	+HCOO, -H
7	0.8	Jionoside D	C30H38O15	683.2214	10,094	+HCOO
8	0.75	2-Acetylacteoside	C31H38O16	665.2115	9,797	-H, +HCOO
9	0.75	Manninotriose	C18H32O16	503.1591	39,609	-H, +HCOO
10	10.27	Benzoyl-oxypaeoniflorin	C30H32O13	599.173	85,386	-H, +HCOO

Notably, several of these abundant constituents—such as albiflorin, poricoic acid G, 7-epi-loganin, and 2-acetylacteoside—have been reported to exert anti-inflammatory or antioxidant activities in previous studies (He, Lau, Xu, Li, & Pui-Hay But, 2000; L. Zhang and Wei, 2020). While their precise roles in disc biology remain to be determined, their identification in the decoction provides a pharmacological basis for subsequent mechanistic investigations of LWDHD in intervertebral disc degeneration.

### 3.2 LWDHD mitigates discogenic pain, preserves endplate integrity, and suppresses ferroptosis-related damage *in vivo*

**FIGURE 2:**
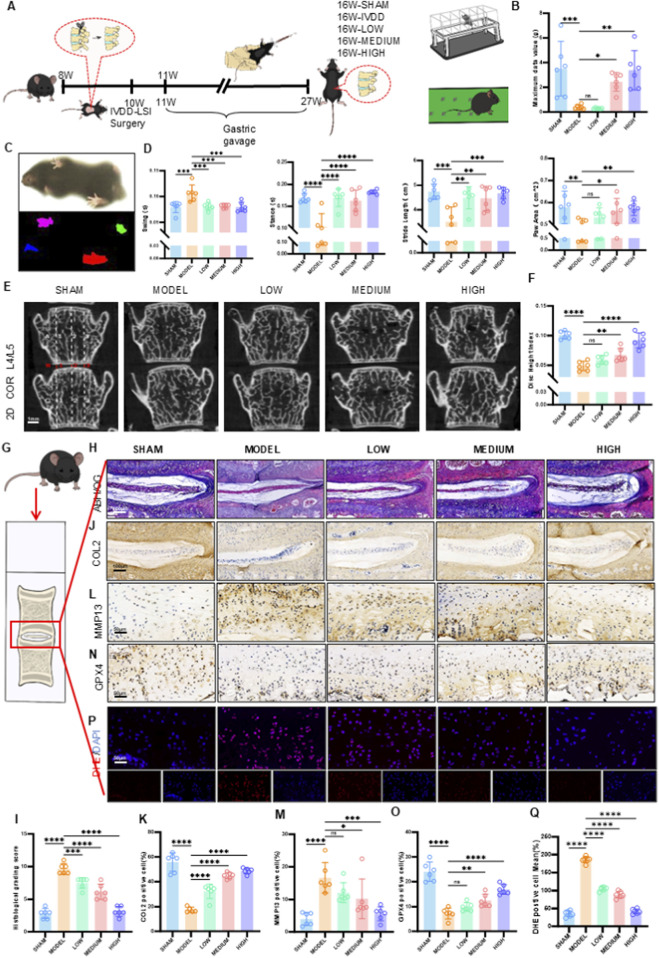
LWDHD alleviates disc degeneration and ferroptosis-related matrix damage *in vivo*. **(A)** *In vivo* workflow, including puncture-induced IVDD, oral LWDHD administration, and outcome assessments. **(B)** Mechanical hypersensitivity measured by von Frey testing (paw withdrawal threshold). **(C,D)** Gait analysis with DigiGait: **(C)** representative frames; **(D)** quantitative parameters (stride length, stance duration, swing time, paw area, print intensity). **(E,F)** Micro-CT evaluation of lumbar discs: **(E)** representative sagittal reconstructions; **(F)** Disc Height Index (DHI) quantification. **(G)** Schematic diagram of mouse spine section. **(H,I)** Alcian Blue–Hematoxylin staining of the endplate region: **(H)** representative images; **(I)** quantification of histological endplate grading score. **(J,K)** Immunohistochemistry (IHC) for type II collagen (COL2) and quantification. **(L,M)** IHC for MMP13 and quantification. **(N,O)** IHC for GPX4 and quantification. **(P,Q)** Dihydroethidium (DHE) staining for reactive oxygen species and fluorescence quantification. Data are mean ± SD (n = 6 animals per group). One-way ANOVA with Tukey’s *post hoc* test. *p < 0.05, **p < 0.01, ***p < 0.001, ****p < 0.0001.

To evaluate the therapeutic efficacy of LWDHD in intervertebral disc degeneration (IVDD), ten-week-old C57BL/6 mice underwent lumbar disc puncture at 11 weeks of age and subsequently received continuous oral administration of LWDHD for 16 weeks (Figure 2A).

Behavioral assessments revealed that LWDHD significantly alleviated discogenic pain. In von Frey tests, mice treated with LWDHD exhibited higher paw withdrawal thresholds compared with untreated IVDD controls (Figure 2B). DigiGait-based analysis demonstrated improvements in stride length, paw area, stance duration, and swing time, indicating functional recovery of motor performance (Figures 2C,D).

Micro-computed tomography (micro-CT) revealed partial preservation of disc height and alignment in LWDHD-treated animals, suggesting macroscopic protection of disc architecture (Figures 2E,F). Histological staining further confirmed enhanced proteoglycan retention in the endplate region, as shown by Alcian Blue–Hematoxylin staining (Figures 2H,I). Immunohistochemistry demonstrated restoration of type II collagen (COL2) and suppression of matrix metalloproteinase 13 (MMP13), consistent with preserved extracellular matrix integrity (Figures 2J–M).

Importantly, ferroptosis-related damage was attenuated. GPX4 expression, which was markedly downregulated in degenerated discs, was restored upon LWDHD treatment (Figures 2N,O). In addition, DHE staining showed reduced ROS accumulation in the endplate region following treatment (Figures 2P,Q). These data indicate that LWDHD mitigates disc degeneration by suppressing ferroptosis-driven matrix damage in EPCs.

Systemic safety was supported by hematoxylin–eosin staining of major organs (heart, liver, spleen, lung, kidney), which revealed no pathological alterations after prolonged LWDHD administration (Supplementary Figure S1).

### 3.3 LWDHD attenuates IL-1β–induced ferroptosis and preserves matrix homeostasis in primary endplate chondrocytes

**FIGURE 3:**
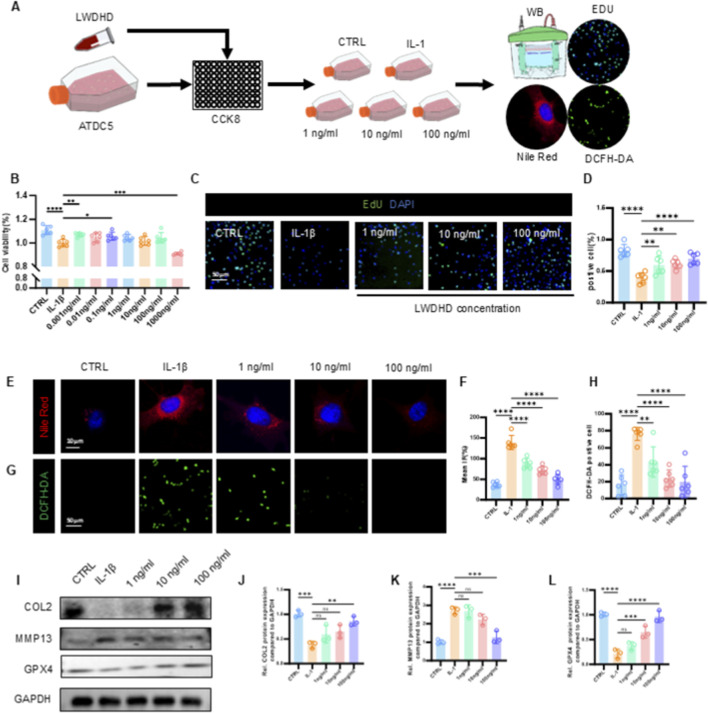
LWDHD suppresses ROS, restores lipid homeostasis, and preserves ECM integrity in IL-1β–stimulated primary endplate chondrocytes. **(A)** Schematic diagram of the *in vitro* workflow. Primary endplate chondrocytes were stimulated with IL-1β and treated with LWDHD solution (1, 10, or 100 ng/mL). **(B)** Cell viability by CCK-8 assay. **(C,D)** EdU staining **(C)** and quantification of EdU-positive nuclei **(D)**. **(E,F)** Nile Red staining for intracellular lipid accumulation **(E)** and quantitative analysis **(F)**. **(G,H)** Intracellular ROS by DCFH-DA staining **(G)** and quantification **(H)**. **(I–L)** Western blot of COL2, MMP13, and GPX4 **(I)** with densitometric quantification normalized to GAPDH **(J–L)**. Data are presented as mean ± SD from five independent experiments. Statistical significance was assessed by one-way ANOVA followed by Tukey’s *post hoc* test. *p < 0.05, **p < 0.01, ***p < 0.001.

To evaluate whether LWDHD exerts direct protective effects on degenerative disc cells, we employed primary endplate chondrocytes as an *in vitro* model. The experimental workflow, including IL-1β stimulation and treatment with LWDHD at concentrations of 1, 10, or 100 ng/mL, is outlined in Figure 3A.

We first performed a dose–response assay using CCK-8 to determine the optimal working concentrations of LWDHD. IL-1β significantly reduced cell viability, while LWDHD restored viability in a dose-dependent manner, with maximal cytoprotective effects observed in the nanogram range (Figure 3B). These concentrations were used in subsequent mechanistic experiments.

EdU staining revealed that IL-1β markedly suppressed chondrocyte proliferation, which was effectively rescued by LWDHD co-treatment, suggesting preserved cell cycle progression under inflammatory stress (Figures 3C,D).

To assess lipid metabolic disturbance, Nile Red staining was used to visualize intracellular lipid accumulation. IL-1β stimulation led to prominent lipid droplet formation, whereas LWDHD significantly reduced lipid content in a concentration-dependent manner (Figures 3E,F).

Intracellular oxidative stress, measured by DCFH-DA fluorescence, was markedly elevated following IL-1β exposure. LWDHD co-treatment dose-dependently suppressed ROS accumulation (Figures 3G,H), suggesting that it mitigates inflammation-induced oxidative damage.

To further investigate ferroptosis, we examined the expression of GPX4 by Western blot. IL-1β significantly downregulated GPX4, consistent with ferroptotic activation, while LWDHD restored GPX4 expression in a dose-dependent fashion (Figures 3I–L). In parallel, LWDHD upregulated COL2 and downregulated MMP13, indicating extracellular matrix preservation under degenerative conditions.

Together, these results confirm that LWDHD directly protects primary endplate chondrocytes from IL-1β–induced ferroptosis by suppressing lipid accumulation and ROS production, restoring GPX4 expression, and preserving matrix protein balance. To further elucidate the molecular pathways underlying these effects, we next performed network pharmacology analysis to systematically explore potential targets and signaling mechanisms of LWDHD in intervertebral disc degeneration.

### 3.4 Network pharmacology analysis reveals TRPA1 as a potential ferroptosis-related target of LWDHD in IVDD

**FIGURE 4:**
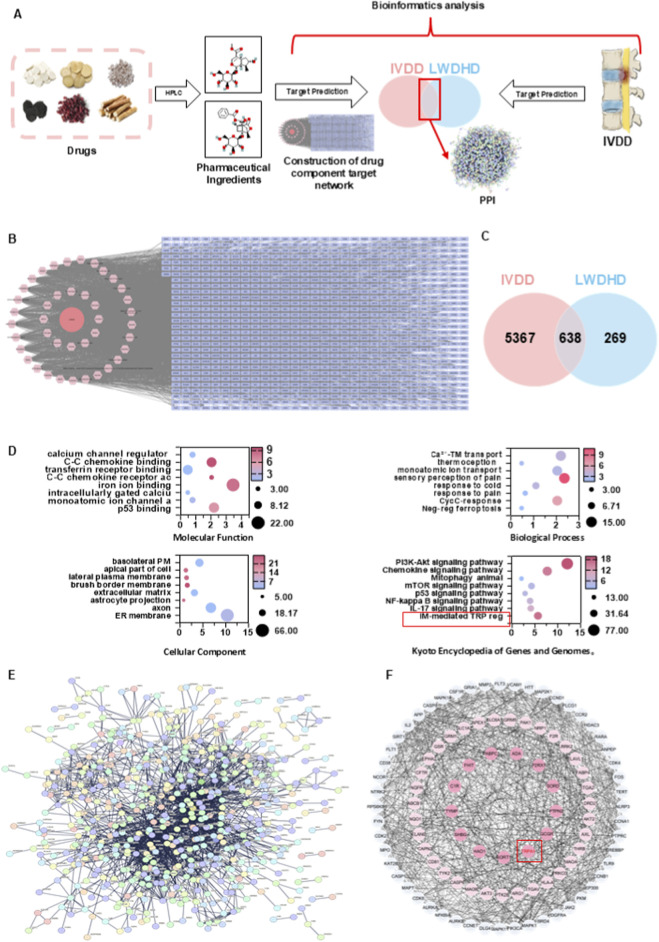
Network pharmacology identifies TRPA1 as a ferroptosis-related target of LWDHD in IVDD. **(A)** Workflow of the integrated network pharmacology analysis combining HPLC profiling, compound–target prediction, and IVDD target mapping. **(B)** Compound–target interaction network: dark-red node = LWDHD, light-red nodes = active compounds, purple squares = predicted target genes. **(C)** Venn diagram showing overlap between IVDD-related targets (n = 6,005) and LWDHD-related targets (n = 907), yielding 638 shared genes. **(D)** GO and KEGG pathway enrichment of overlapping targets. **(E)** Protein–protein interaction (PPI) network of the 638 shared targets. **(F)** Hub gene analysis highlighting TRPA1 and other key regulators.

To elucidate the potential mechanistic targets of LWDHD in IVDD, we conducted an integrated network pharmacology analysis (Figure 4A). A total of 907 putative targets were identified from LWDHD-related compounds, among which 638 genes overlapped with 6005 IVDD-related targets retrieved from GeneCards (Figures 4B,C).

Gene Ontology (GO) enrichment of these 638 intersecting genes revealed strong associations with biological processes and molecular functions involved in calcium ion transmembrane transport, negative regulation of ferroptosis, iron ion binding, monoatomic ion channel activity, C–C chemokine receptor activity, C–C chemokine binding, p53 binding, and calcium channel regulation, many of which have been implicated in ferroptotic and inflammatory processes in degenerative joint disease (Figure 4D).

KEGG pathway enrichment further emphasized PI3K-Akt, chemokine signaling, mitophagy, mTOR, p53, NF-κB, and IL-17 signaling pathways. Importantly, the term “inflammatory mediator regulation of TRP channels” emerged as one of the top-ranked pathways, highlighting the TRP ion channel family, including TRPA1, as potential mediators linking inflammation and ferroptosis in IVDD.

To explore the core regulatory hubs, we constructed a protein–protein interaction (PPI) network of the overlapping genes (Figure 4E). Topological analysis identified several key hub genes based on node degree centrality, including TRPA1, ADA, P2RX7, SORD, PTPN2, GCG, SORT1, and HAO1 (Figure 4F). Among these, TRPA1 ranked prominently, supporting its relevance as a potential ferroptosis-associated target involved in the calcium signaling–inflammation axis in degenerative disc disease.

Taken together, these findings suggest that TRPA1 may serve as a critical intermediary through which LWDHD exerts anti-ferroptotic and anti-inflammatory effects in IVDD. In the next section, we experimentally evaluate the functional role of TRPA1 in degenerative disc cells and validate whether LWDHD confers protection through TRPA1 suppression.

### 3.5 LWDHD confers protection against ferroptosis and matrix degradation through TRPA1 modulation

**FIGURE 5:**
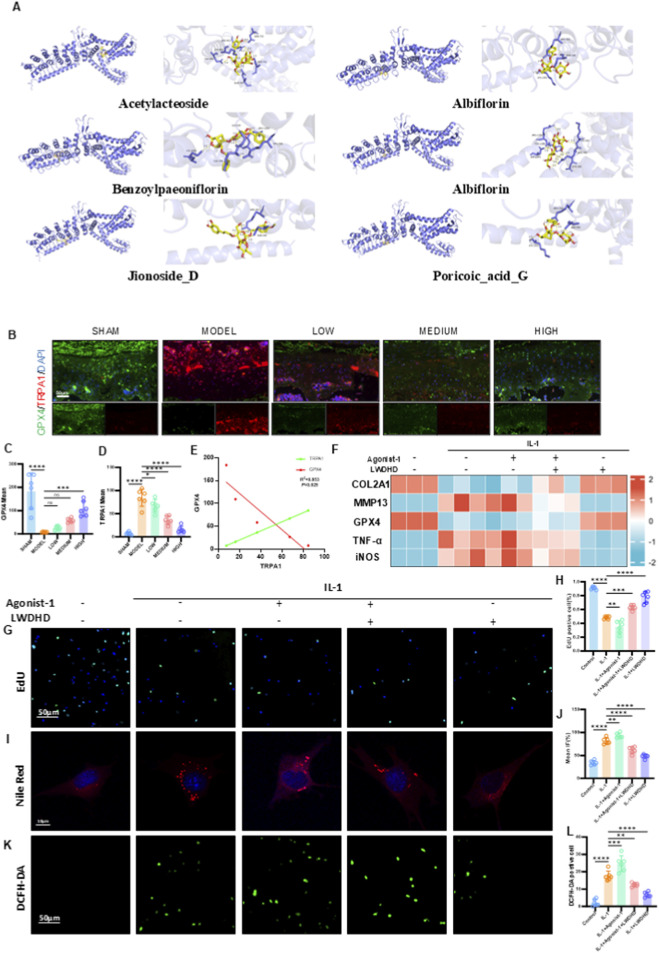
TRPA1 mediates the protective effects of LWDHD against ferroptosis and ECM degradation. **(A)** Molecular docking of representative LWDHD compounds (Acetylacteoside, Albiflorin, Benzoylpaeoniflori, Gentianose, Jionoside_D,Poricoic_acid_G) with TRPA1. Ligands are shown as yellow sticks in the binding pocket. **(B)** Immunofluorescence staining of GPX4 (green) and TRPA1 (red) in disc tissues from control, model, and LWDHD-treated groups (low, medium, high). Nuclei counterstained with DAPI (blue). **(C,D)** Quantification of GPX4 (c) and TRPA1 (d) fluorescence intensity. **(E)**. Correlation analysis between GPX4 and TRPA1. Rescue experiments using Agonist-1 (TRPA1 agonist) in primary endplate chondrocytes. Groups: control, IL-1β, IL-1β + Agonist-1, IL-1β + Agonist-1 + LWDHD, IL-1β + LWDHD. **(F)** RT-qPCR of COL2A1, MMP13, GPX4, TNF-α, iNOS. **(G,H)** EdU staining and quantification. **(I,J)** Nile Red staining and quantification. (K–L) DCFH-DA fluorescence for ROS and quantification. Data are presented as mean ± SD (n = 6 animals per group for *in vivo* experiments; n = 5 independent replicates for *in vitro* experiments). Statistical significance was assessed by one-way ANOVA followed by Tukey’s *post hoc* test. *p < 0.05, **p < 0.01, ***p < 0.001, ****p < 0.0001.

To validate the functional role of TRPA1 in mediating the effects of LWDHD, molecular docking was first performed. Several representative compounds of LWDHD, including morroniside, loganin, paeoniflorin, alisol B, and catalpol, exhibited stable binding interactions within the TRPA1 channel pocket, supporting the plausibility of direct modulation (Figure 5A). The detailed docking parameters—including binding energy, degree of conformational change after substrate docking—are summarized in Table 3.

**TABLE 3:** Molecular docking details information.

Component name	Affinity (kcal/mol)	Dist from best mode	
		rmsd l.b	rmsd u.b
**2_Acetylacteoside**	**−7.4**	**2.340**	**4.561**
3beta_Hydroxylanosta	−6.3	26.397	29.601
7_epi_Loganin	−6.8	23.292	25.963
**Albiflorin**	**−7.7**	**39.740**	**43.596**
**Benzoylpaeoniflorin**	**−8.3**	**37.438**	**40.156**
16-Oxoalisol A	−4.6	25.440	27.211
**Gentianose**	**−7.0**	**41.472**	**45.323**
**Jionoside_D**	**−8.0**	**41.141**	**48.231**
Manninotriose	−6.5	2.856	7.155
**Poricoic_acid_G**	**−6.8**	**10.463**	**12.709**

The bolded parts in the table represent components with binding energy < -6.5.

*In vivo* immunofluorescence analysis revealed an inverse relationship between GPX4 and TRPA1 in intervertebral disc tissues. Compared with control discs, degenerated tissues showed decreased GPX4 and elevated TRPA1, while LWDHD treatment dose-dependently restored GPX4 and reduced TRPA1 expression (Figures 5B–D). Correlation analysis confirmed a significant negative association between GPX4 and TRPA1 (Figure 5E).

To further establish causality, we performed rescue experiments using Agonist-1, a selective TRPA1 agonist, in primary endplate chondrocytes. As expected, IL-1β stimulation markedly reduced COL2A1 and GPX4 while increasing MMP13, TNF-α, and iNOS expression. Treatment with LWDHD effectively reversed these molecular changes; however, Agonist-1 not only exacerbated the IL-1β-induced detrimental effects but also abolished the restorative actions of LWDHD (Figure 5F).

At the functional level, EdU staining revealed that IL-1β suppressed cell proliferation, which was rescued by LWDHD but negated by Agonist-1 co-treatment (Figures 5G,H). Nile Red staining showed that IL-1β promoted lipid accumulation, which was attenuated by LWDHD but reinstated when TRPA1 was activated by Agonist-1 (Figures 5I,J). Similarly, ROS levels measured by DCFH-DA fluorescence were suppressed by LWDHD yet remained elevated under combined Agonist-1 and LWDHD treatment (Figures 5K,L).

Collectively, these findings demonstrate that LWDHD protects against ferroptosis and matrix degradation in degenerative EPCs by modulating TRPA1 activity. Importantly, pharmacological activation of TRPA1 abolishes the protective effects of LWDHD, underscoring TRPA1 as a critical downstream target through which LWDHD confers anti-ferroptotic and matrix-preserving actions.

### 3.6 *In vivo* rescue experiments confirm that the protective effects of LWDHD depend on TRPA1 modulation

**FIGURE 6:**
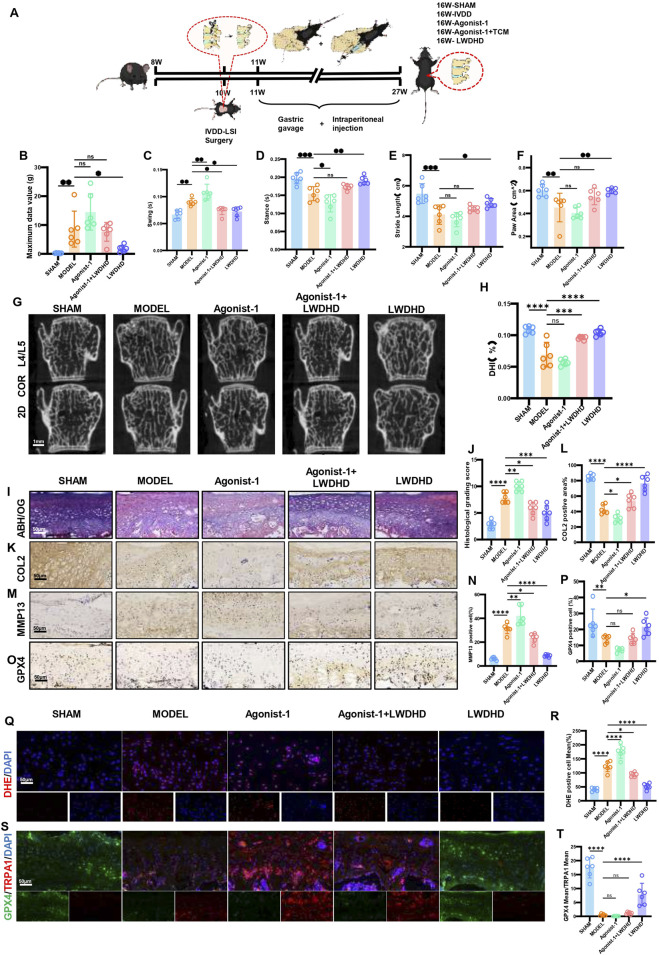
*In vivo* rescue experiments confirm that LWDHD protection depends on TRPA1 modulation. **(A)** Experimental workflow in the mouse puncture-induced IDD model, including five groups: Sham, Model, Agonist-1, Agonist-1 + LWDHD, LWDHD. **(B)** Von Frey testing for mechanical allodynia. **(C–F)** Gait analysis: swing time **(C)**, stance time **(D)**, stride length **(E)**, paw area **(F)**. **(G,H)** Representative micro-CT images **(G)** and Disc Height Index (DHI) quantification **(H)**. **(I,J)** ABH/OG histological staining **(I)** and scoring **(J)**. **(K,L)** IHC for Col2a1 and quantification. **(M,N)** IHC for Mmp13 and quantification. **(O,P)** IHC for GPX4 and quantification. **(Q,R)** DHE fluorescence for ROS and quantification. **(S,T)** IF staining of GPX4 (green) and TRPA1 (red) with DAPI **(S)**, and GPX4/TRPA1 fluorescence ratio **(T)**. Data are presented as mean ± SD (n = 6 animals per group). Statistical significance was assessed by one-way ANOVA followed by Tukey’s post hoc test. *p < 0.05, **p < 0.01, ***p < 0.001, ****p < 0.0001.

To further validate the role of TRPA1 in mediating LWDHD’s therapeutic benefits, we performed rescue experiments in the puncture-induced IVDD mouse model. Animals were divided into five groups: Sham, Model, Agonist-1, Agonist-1 + LWDHD, and LWDHD (Figure 6A).

Behavioral tests showed that LWDHD significantly reduced discogenic pain, as evidenced by higher paw withdrawal thresholds in von Frey assays and improved gait parameters, including stride length, stance duration, swing time, and paw area (Figures 6B–F). However, these effects were abrogated when TRPA1 was activated by Agonist-1, suggesting that LWDHD’s analgesic effects require TRPA1 suppression.

Structural assessment by micro-CT and Disc Height Index (DHI) analysis confirmed that LWDHD preserved disc height and alignment, whereas Agonist-1 reversed these protective outcomes (Figures 6G,H). Histological staining (ABH/OG) demonstrated proteoglycan retention in the endplate region after LWDHD treatment, but this benefit was lost with TRPA1 activation (Figures 6I,J).

At the molecular level, immunohistochemical analyses revealed that LWDHD upregulated COL2a1 and GPX4 while downregulating MMP13, consistent with ECM preservation and ferroptosis inhibition. These molecular effects were abolished in the presence of Agonist-1 (Figures 6K–P). Similarly, DHE fluorescence staining confirmed reduced ROS accumulation in LWDHD-treated discs, whereas ROS remained elevated when TRPA1 was activated (Figures 6Q,R). Immunofluorescence double staining further demonstrated that LWDHD restored GPX4 expression while suppressing TRPA1, but these effects were negated by Agonist-1 co-treatment (Figures 6S,T).

Together, these results provide strong *in vivo* evidence that LWDHD protects against IVDD through modulation of TRPA1. Pharmacological activation of TRPA1 not only exacerbates degeneration but also abolishes LWDHD’s analgesic, structural, and molecular benefits, underscoring TRPA1 as an indispensable downstream mediator of LWDHD efficacy.

## 4 Discussion

IVDD is increasingly recognized as a multifactorial disorder involving inflammation, oxidative stress, and ECM breakdown (Chen et al., 2024; Zhao et al., 2025). Among emerging mechanisms, ferroptosis has been identified as a distinct form of regulated cell death that contributes to disc cell depletion and progressive degeneration (Fan et al., 2023). In the present study, we provide evidence that TRPA1 is a critical regulator of ferroptosis in endplate chondrocytes and demonstrate that LWDHD, a classical herbal formula, attenuates IVDD by modulating TRPA1 signaling (Ye L. et al., 2025; Zhong et al., 2025).

Our findings extend current understanding of ferroptosis in disc pathology. While previous studies have emphasized the role of glutathione depletion and GPX4 inactivation in driving ferroptotic sensitivity, our results highlight the contribution of TRPA1, a Ca^2+^-permeable ion channel traditionally studied in nociception and inflammatory responses (Lin et al., 2025). Previous studies have shown that there are significant differences in cell-cell interactions and multiple mechanism pathways between normal intervertebral disc cells and those from patients with IVDD (Li et al., 2022; Ye Y. et al., 2025). Lin’s wound research proposes a molecular mechanism pathway that regulates downstream inflammatory factor activity by influencing TRPA1 activity to induce Ca^2+^ flow, promoting GPX4 elevation, and subsequently inhibiting ferroptosis (Lin et al., 2025). We observed that TRPA1 expression was markedly elevated in degenerative discs, inversely correlating with GPX4 levels. Pharmacological activation of TRPA1 using Agonist-1 not only exacerbated oxidative stress and lipid peroxidation but also abolished the protective effects of LWDHD, both *in vitro* and *in vivo*. These findings identify TRPA1 as a novel driver of ferroptosis in disc cells and suggest that its inhibition may represent a therapeutic avenue for IVDD (Wang et al., 2024; Yurube, Takeoka, Kanda, Kuroda and Kakutani, 2023).

LWDHD demonstrated broad benefits at the structural, cellular, and behavioral levels. *In vivo* administration alleviated mechanical hypersensitivity, improved gait parameters, preserved disc height, and restored proteoglycan content. At the molecular level, LWDHD restored GPX4 expression, reduced ROS accumulation, suppressed lipid droplet formation, and preserved ECM proteins such as COL2 while downregulating MMP13. *In vitro*, LWDHD rescued IL-1β–stimulated chondrocytes from ferroptotic death, normalized redox balance, and promoted proliferative activity. These pleiotropic effects are consistent with the multi-component nature of LWDHD, which, according to our docking and network pharmacology analyses, contains constituents capable of interacting with TRPA1 and other ferroptosis-related targets (Ge et al., 2024; Zhang et al., 2021).

The identification of TRPA1 as a downstream mediator of LWDHD also provides a mechanistic explanation for the analgesic effects observed. TRPA1 is well known for its role in sensory transduction and pain hypersensitivity (Koivisto, Jalava, Bratty and Pertovaara, 2018; Souza Monteiro de Araujo, Nassini, Geppetti and De Logu, 2020). By suppressing TRPA1 activation, LWDHD not only reduced ferroptotic stress but also alleviated discogenic pain. This dual effect underscores the clinical potential of TRPA1 modulation as a strategy to address both structural and symptomatic aspects of disc degeneration (Liu et al., 2019; Xie, Wang and Gong, 2025).

From a translational perspective, these findings provide molecular evidence supporting the traditional use of LWDHD in musculoskeletal disorders. In TCM theory, LWDHD is prescribed to “tonify the kidney and nourish Yin,” functions historically associated with spinal stability and skeletal health. Our study bridges this classical concept with modern molecular mechanisms, showing that LWDHD protects disc tissues by targeting oxidative stress and ferroptotic death through TRPA1 modulation. This mechanistic rationale strengthens the case for clinical application of LWDHD as a multi-target therapy in IVDD (Chen et al., 2022; Samanta, Lufkin and Kraus, 2023; Zhou C. et al., 2022).

Nevertheless, several limitations must be acknowledged. First, our mechanistic conclusions are largely based on pharmacological modulation of TRPA1. Genetic approaches, such as TRPA1 knockout or conditional knockdown models, would provide more definitive causal evidence. Second, the precise contributions of individual LWDHD compounds remain to be clarified. Although docking analyses identified stable interactions between TRPA1 and multiple bioactive constituents, fractionation and bioactivity-guided assays are necessary to confirm which molecules directly modulate TRPA1 activity *in vivo*. Finally, while our murine models reproduce key features of human disc degeneration, validation in human tissues and clinical studies will be required to establish translatability (Yan et al., 2022).

## Data Availability

The datasets presented in this study can be found in online repositories. The names of the repository/repositories and accession number(s) can be found in the article/Supplementary Material.
