# Supplementary material for: Liuwei dihuang decoction attenuates intervertebral disc degeneration by inhibiting TRPA1-Mediated ferroptosis in endplate chondrocytes
Source: Front Cell Dev Biol. 2026 May 29;14:1759798. doi: 10.3389/fcell.2026.1759798 (PMC13260549; doi:10.3389/fcell.2026.1759798)
Supplement: Supplementary file 1 [file Table1.docx]

**Table 1** Composition and origin of LWDHD

| **Pharmaceutical name** | ***Botanical plant names***  **and authorities** | **Traditional medicinal parts** | **Origin** | **Weight (g)** |
| --- | --- | --- | --- | --- |
| Shu dihuang | *Rehmannia glutinosa* (Gaertn.) DC. | root tuber | Henan | 15 |
| Shan zhuyurou | *Cornus officinalis* Siebold & Zucc. | pulp | Zhejiang | 12 |
| Shanyao | *Dioscorea polystachya* Turcz. | root tuber | Zhejiang | 12 |
| Mu danpi | *Paeonia × suffruticosa* Andrews | root bark | Anhui | 10 |
| Zexie | *Alisma plantago-aquatica* L. | root tuber | Sichuang | 10 |
| Fuling | *Wolfiporia cocos* (F.A. Wolf) Ryvarden & Gilb. | Sclerotia | Zhejiang | 10 |

**Table 2** The detailed HPLC-MS parameters of representative compounds

| **Peak**  **No.** | **Observed RT（min）** | **Component name** | **Formula** | **Observed m/z** | **Response** | **Adduct** |
| --- | --- | --- | --- | --- | --- | --- |
| 1 | 6.15 | 7-epi-Loganin | C17H26O10 | 413.1433 | 6001 | +Na, +H, +K |
| 2 | 13.7 | Poricoic acid G | C30H46O5 | 487.3426 | 6515 | +H |
| 3 | 12.16 | 3beta-Hydroxylanosta-7,9 (11),24-trien-21-oic acid | C30H46O3 | 455.353 | 6030 | +H |
| 4 | 0.78 | Gentianose | C18H32O16 | 505.1783 | 5072 | +H |
| 5 | 13.7 | 16-Oxoalisol A | C30H48O6 | 505.3525 | 35617 | +H, +Na |
| 6 | 6.75 | Albiflorin | C23H28O11 | 525.1595 | 259628 | +HCOO, -H |
| 7 | 0.8 | Jionoside D | C30H38O15 | 683.2214 | 10094 | +HCOO |
| 8 | 0.75 | 2-Acetylacteoside | C31H38O16 | 665.2115 | 9797 | -H, +HCOO |
| 9 | 0.75 | Manninotriose | C18H32O16 | 503.1591 | 39609 | -H, +HCOO |
| 10 | 10.27 | Benzoyl-oxypaeoniflorin | C30H32O13 | 599.173 | 85386 | -H, +HCOO |

**Table 3** Molecular docking details information

| **Component name** | **Affinity**  **(kcal/mol)** | **dist from best mode** | |
| --- | --- | --- | --- |
|  |  | **rmsd l.b.** | **rmsd u.b.** |
| **2_Acetylacteoside** | **-7.4** | **2.340** | **4.561** |
| 3beta_Hydroxylanosta | -6.3 | 26.397 | 29.601 |
| 7_epi_Loganin | -6.8 | 23.292 | 25.963 |
| **Albiflorin** | **-7.7** | **39.740** | **43.596** |
| **Benzoylpaeoniflorin** | **-8.3** | **37.438** | **40.156** |
| 16-Oxoalisol A | -4.6 | 25.440 | 27.211 |
| **Gentianose** | **-7.0** | **41.472** | **45.323** |
| **Jionoside_D** | **-8.0** | **41.141** | **48.231** |
| Manninotriose | -6.5 | 2.856 | 7.155 |
| **Poricoic_acid_G** | **-6.8** | **10.463** | **12.709** |

**Supplementary Table 1** Detailed information of primary antibodies

| **Antibody** | **Cat. No.** | **Dilution** | **Application** |
| --- | --- | --- | --- |
| COL2 | Proteintech, 28459-1-AP | 1:1000 | For IHC |
| MMP13 | Proteintech, 18165-1-AP | 1:500 | For IHC |
| GPX4 | Proteintech, 30388-1-AP | 1:500 | For IHC |
| GPX4 | Proteintech, 30388-1-AP | 1:500 | For IF |
| TRPA1 | Proteintech, 19124-1-AP | 1:1000 | For IF |
| COL2 | Proteintech, 28459-1-AP | 1:1000 | For WB |
| MMP13 | Proteintech, 18165-1-AP | 1:2000 | For WB |
| GPX4 | Proteintech, 30388-1-AP | 1:1000 | For WB |
| GAPDH | Abclonal, A19056 | 1:5000 | For WB |

**Supplementary Table 2** **RNA primer sequence** (5’-3’)

| **mRNA** | **Forward** | **Reverse** |
| --- | --- | --- |
| COL2A1 | GCTGGTGAAGAAGGCAAACGAG | CCATCTTGACCTGGGAATCCAC |
| MMP13 | GATGACCTGTCTGAGGAAGACC | GCATTTCTCGGAGCCTGTCAAC |
| GPX4 | CCTCTGCTGCAAGAGCCTCCC | CTTATCCAGGCAGACCATGTGC |
| TNF-α | GGTGCCTATGTCTCAGCCTCTT | GCCATAGAACTGATGAGAGGGAG |
| INOS | GAGACAGGGAAGTCTGAAGCAC | CCAGCAGTAGTTGCTCCTCTTC |
