# Supplementary figures and images for: Liuwei dihuang decoction attenuates intervertebral disc degeneration by inhibiting TRPA1-Mediated ferroptosis in endplate chondrocytes
Source: Front Cell Dev Biol. 2026 May 29;14:1759798. doi: 10.3389/fcell.2026.1759798 (PMC13260549; doi:10.3389/fcell.2026.1759798)

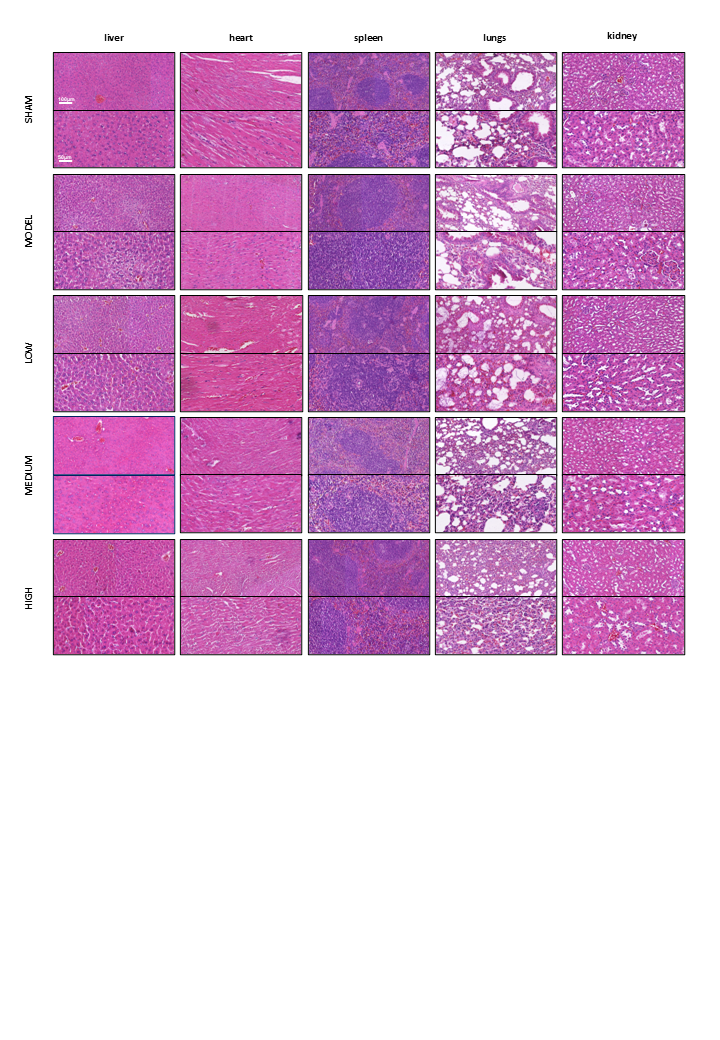

Supplement: Supplementary file 2 [file Image1.tif]
